# The effect of group-dynamics, collaboration and tutor style on the perception of profession-based stereotypes: a quasi-experimental pre- post-design on interdisciplinary tutorial groups

**DOI:** 10.1186/s12909-021-02814-5

**Published:** 2021-07-10

**Authors:** Eva Hammar Chiriac, Endre Sjøvold, Alexandra Björnstjerna Hjelm

**Affiliations:** 1 Department of Behavioral Sciences and Learning, Linköping University, SE-581 83 Linköping, Sweden; 2 Department of Industrial Economics and Technology Management, Norwegian University of Science and Technology, NTNU, Trondheim, Norway

**Keywords:** Interprofessional collaboration, Group-dynamics, Interprofessional problem-based learning (iPBL), Spin theory, SPGR, Observation, Professional stereotypes, Tutor style

## Abstract

**Background:**

Group processes in inter-professional Problem-Based Learning (iPBL) groups have not yet been studied in the health-care educational context. In this paper we present findings on how group-dynamics, collaboration, and tutor style influence the perception of profession-based stereotypes of students collaborating in iPBL groups. Health-care students are trained in iPBL groups to increase their ability to collaborate with other healthcare professionals. Previous research focusing iPBL in healthcare implies that more systematic studies are desired, especially concerning the interaction between group processes and internalized professional stereotypes. The aim of this study is to investigate whether changes in group processes, collaboration, and tutor style, influence the perception of profession-based stereotypes of physician- and nursing-students.

**Methods:**

The study is a quasi-experimental pre- post-design. The participants included 30 students from five different healthcare professions, mainly medicine and nursing. Other professions were physiotherapy, occupational therapy and speech therapy. The students were divided into four iPBL groups, each consisting of six to nine students and a tutor. Data were collected through systematic observation using four video-recorded tutorials. SPGR (Systematizing the Person Group Relation), a computer-supported method for direct and structured observation of behavior, was used to collect and analyze the data.

**Results:**

Traditional stereotypical profession-based behaviors were identified in the first observed group meeting. Although the groups followed different paths of development, the group-dynamics changed in all groups over the 6 weeks of collaboration. Two of the groups became more cohesive, one became more fragmented and one became more polarized. Stereotypical behaviors became less frequent in all groups. Our findings indicate that tutor behavior has a strong influence on the development of the group’s dynamics.

**Conclusion:**

Our findings strongly suggest iPBL is a means of reducing stereotypical behaviors, and may positively increase members’ ability to engage in inter-professional collaboration. Although the pattern of dynamics took different forms in different groups, we argue that iPBL forces students to see the colleague behind his or her profession, thus breaking professional boundaries. The tutor style significantly influenced the iPBL groups’ development. This study contributes to our field by emphasizing the effect of group-processes in increasing mutual understanding across professions.

## Background

The use of inter-professional Problem-Based Learning (iPBL) groups in healthcare education is used as a means to increase collaboration among pre-qualified students from different healthcare specializations. IPBL, an approach aligning problem-based learning (PBL) [1–3] with Interprofessional Education (IPE) [4–6], can be defined as curricular activities in which student groups from different health professional programs “learn from, with and about each other to improve collaboration and the quality of care” [5, 7–10]. Such interactions between students are intended to create training synergies and collaboration skills in heterogenous groups of healthcare students and to facilitate a better understanding of other professions [5, 6, 11, 12]. Freeth and colleagues [5] emphasize the need to distinguish between (a) learning *about*, for instance acquiring an understanding of other professional roles and how to reduce stereotypical associations, (b) learning *with*, which is team work and collaboration and (c) learning *from*, which is connected to trust, respect, and confidence in the competence of other professionals.

Researchers studying pre-qualified students in healthcare education seem to agree on the efficacy of both PBL and IPE and that there is a strong incentive for its using iPBL in healthcare education [4–6]. IPBL facilitates the acquisition of collaborative knowledge, skills and attitudes, enabling collaborative practice [13]. In a review including 83 studies between 2005 and 2010, Abu-Risch and colleagues [4] concluded that research about IPE in healthcare education has reported primarily on four major themes, partly confirmed by complementary research; a) *students’ learning* [9, 14], b) *professional roles/identification* [15, 16], c) *communication* [17–19], and d) *overall satisfaction with IPE* [6, 14]. However, we would like to add another theme: e) *implementation and development* [6, 20, 21]. Still, studies on IPE, and thus also iPBL, group-processes in general, and groups’ development specifically are lacking; this is thus a research gap requiring further research [19, 22].

In order to improve security and sustain safe and effective healthcare, developing interprofessional collaboration between pre-qualified students from an increasing number of healthcare professions, using iPBL [23], is an absolute requisite [9, 12, 13, 24, 25]. Previous studies have shown that professional stereotypes may be a barrier to collaboration both in education [15, 19, 26, 27] and in practice [28, 29]. Being a member of a profession is a source of professional identity and confidence in an interdisciplinary context. As such, professional stereotypes are not only held by outsiders, but also reinforced by the professionals themselves [29]. Research on the perception of profession-based stereotypes of physician- [18, 30] and nursing- students [31] have received the most attention. Nurses are described as warm, empathic and caring, often acting submissive, whereas physicians are seen as independent, active, and domineering, all traits that reflect the traditional roles or stereotypes [15, 17, 18]. Furthermore, studies have consistently shown that profession-based stereotypes are persistent [29, 32–34] and even influence students before they have entered healthcare education [15, 26]. Thylefors [27] went so far as to argue that, at the beginning of their education, medical students already identify with their future professions and state that prejudices about different professions influence their choice of education. A conclusion later confirmed by Athea et al. [26] and Pietroni [34] revealed that students provided stereotypical images of their own and others’ professions.

The fundamental idea behind iPBL is that the group processes are the main vehicle of learning, carried out, applied, and implemented in interactions with other students in a tutorial group lead by a tutor [3, 19, 22]. PBL being a student-centered approach that promotes self-directed learning, problem solving and group processes as a means for learning is naturally suitable for IPE [4, 5]. In iPBL, active contribution to the group’s joint work is mandatory and is facilitated by vignettes enabling collaborative practice [5, 9, 11].

Even though research has not been able to provide the characteristics of effective tutoring in either PBL or iPBL [3, 11, 19, 35, 36], researchers seem to agree that the tutors should behave in accordance with the philosophy of PBL [3, 19, 37, 38] by facilitating the process rather than delivering content. The characteristics of effective tutoring are not clear-cut, and tutor style is best negotiated and developed through interactions with each tutorial group, to promote successful group development. The tutor exerts more control at the beginning of the group’s life [3, 11, 19, 35, 36]; their role and behavior adapts over time to the group’s development and to the context (i.e., the task and group composition).

The aspects of group-dynamics [19, 22] and leadership [5, 11] in iPBL is highly understudied. Recent research emphasizes that different contexts demand different dynamics for a group to be effective [39–43]. In spin theory [43, 44] the construct balance is used to explain how group dynamics may change to suit contextual demands. In a stable context with simple tasks, a group may be effective with a fixed role-structure and strong leadership, but with increased complexity and ambiguous tasks, a more flexible role structure is needed. One would expect an iPBL group to develop a flexible role-structure to be successful since the learning situation demands shared situational awareness and equal contributions from the group-members from different health-care educations and professions.

The operationalization of the spin theory is labelled SPGR (Systematizing the Person Group Relation) and is a structured method for observing group behavior, including algorithms for analyzing distribution of influence, polarization, degree of opposition, and other important aspects of group-dynamics. SPGR has been developed and refined over the last 40 years based on the observations of thousands of groups [29, 43, 45–49]. The Norwegian Armed Forces have used SPGR in their training of military groups for more than 20 years.

### Purpose

The purpose of this study was to investigate whether changes in group processes, collaboration and tutor style influence the perception of profession-based stereotypes of physician- and nursing-students. This leads to the following research questions:

1. ‘What professional stereotypes are perpetuated between physician- and nursing-students during the initial iPBL groups? What kind of changes, if any, appear in the dynamics of the iPBL groups and the group-members’ perception of professional stereotypes?
2. How does the tutor’s behavior influence the group’s development and collaboration?

## Methods

### Design

The study was designed as a quasi-experimental pre-post study in which the groups are videotaped and observed at the start of the group sessions and at their final meeting. Each tutorial group consisted of a tutor and a mixture of six to nine students of medicine and nursing and from other areas of healthcare education such as physiotherapy, occupational therapy, and speech therapy (Table 1).

**Table 1:** Distribution of students’ choice of healthcare profession in the four tutorial groups

Profession:	Tutorial group 1	Tutorial group 2	Tutorial group 3	Tutorial group 4
Physician	3	3	3	3
Nurse	3	2	2	3
Physiotherapist		1	1	1
Occupational therapist	1	1		1
Speech therapist		1		1
Total	7	8	6	9

All tutorial groups included in this study were natural groups in an authentic situation. We used direct observation to investigate the development of dynamics in four tutorial groups. We based our observations on video recordings of two group sessions separated by a six-week interval and used SPGR-software to directly observe group behavior and to map and analyze our data using a validated category system [43, 47].

### Participants

The participants came from a cohort of 400 healthcare students at a large university in a medium-sized Swedish city during the second semester of their professional education, hence all students had at least one term’s experience of PBL. All students and tutors who participated in the mandatory six-week course in interdisciplinary collaboration were informed of, and invited to participate in, the study. A total of 35 people from four tutorial groups agreed to participate in this study. Thirty of the 35 were students (21 women and 9 men) between 19 and 40 years of age and five were tutors (all female). The randomization of allocation to each tutorial group was administered by the medical department responsible for the course. All tutors belonged to a healthcare profession and were experienced tutors, having acted as tutors in PBL before. They had also undergone compulsory college pedagogical tutorial group education.

### Data collection

The four tutorial groups were video-recorded using three to four cameras in each session. The cameras were up and running before the session started. The researcher was not present in the room during the sessions. During the sessions, all tutorial groups worked with the same vignettes, developed to suit iPBL. In accordance with Freeth and colleagues’ [5] recommendations, all vignettes were carefully selected to ensure that all group members, irrespective of professional affiliation, could contribute to the common work, and to facilitate collaborative learning among the students. We observed the first and the last sessions of each group. In each session, we observed a) the beginning, b) the choice of problem formulation (approximately the middle of the session) and c) the session evaluation, which was the last activity. Thus, the observations spanned a period of time both between and within sessions, allowing us to get a fair picture of the development of behavior in the tutorial group. In total, this study builds on observations of four-hours of video-recordings.

#### The SPGR system for direct observations of group behavior

In the SPGR system, behaviors supporting the four group functions are grouped in 12 categories (See Table 2) [46–48]. Direct observation of groups using SPGR is supported by touchscreen-based software by which a trained observer registers a) which person acts and towards whom, b) what behavioral category best describes the act and c) whether the act was verbal or non-verbal. Hence, each observation included three decisions.

**Table 2:** Overview of the SPGR system for direct observation of group behavior [46, 47]. The four first categories C, N, O and D cover behavior supporting the four basic group functions. Category W covers behavior that may lead to dissolution of the group and category S covers behavior that enhances collaboration

Main category *Sub-categories*	Typical behavior	Body language
**Control (C) (Task-orientation)**		
*Ruling (C1)*	Controlling, authoritarian, emphasize rules and procedures.	Demanding, using eye-contact to control.
*Task-oriented (C2)*	Objective, efficient, analytical.	Neutral, slightly formal.
**Nurture (N) (Relation-orientation)**		
*Caring (N1)*	Empathic, thoughtful, sociable.	Open expressive using eye-contact to recognize.
*Spontaneous (N2)*	Creative, spontaneous, emotional.	Large gestures.
**Opposition (O) (Opposing)**		
*Criticism (O1)*	Provocative, oppositional, non-conformal.	May appear aggressive.
*Assertiveness (O2)*	Competitive, stubbornly, self-confident.	Dominating, rejecting.
**Dependence (Obligation-oriented)**		
*Loyalty (D1)*	Passive, obedient, dutiful.	Submissive, small gestures.
*Acceptance (D2)*	Trusting, confident, satisfied.	Nice, show acceptance of the group.
**Withdrawal (W) (Dissolution)**		
*Resignation (W1)*	Dejected, lack of will to contribute and cooperate.	Detached, rejecting and closed.
*Selfish (W2)*	Sad, withdrawn, offended, accusing.	Closed and tense, displaying ignorance.
**Synergy (S) (Teamwork)**		
*Engagement (S1)*	Collaborative, inclusive, encouraging.	Active and lively, inviting, direct eye contact
*Empathy (S2)*	Listening, empathetic, supportive	Lively, inviting and confirming, seeking eye contact.

Both observers had previous experience of using group observation as a method for data collection. The first author, who was one of the observers, had more than 25 years of experience in using group observation as a scientific method, had published several articles (e.g., [19]) using direct observation as a means for data collection, written a textbook on group observations as a scientific method [46] and conducted university courses at both the undergraduate and postgraduate levels on how to use observations with both low and high structure as a scientific method. The other observer had received training and conducted previous studies using group observation as a method for data collection. Both observers are certified users of SPGR and thereby passed the reliability test (see below). Together, the two observers are knowledgeable and previous experience in conducting credible group observations of high quality.

The SPGR observation system is based on a validated category system operationalized with a high degree of structure from the spin theory of groups [44, 48]. The observers were trained over a six-month period and passed a reliability test. The reliability test is standardized and performed by letting the candidate observe a videotaped group-interaction. The candidate’s results should be within previously determined deviations from experts’ observations of the same group interaction.

**Fig. 1:**
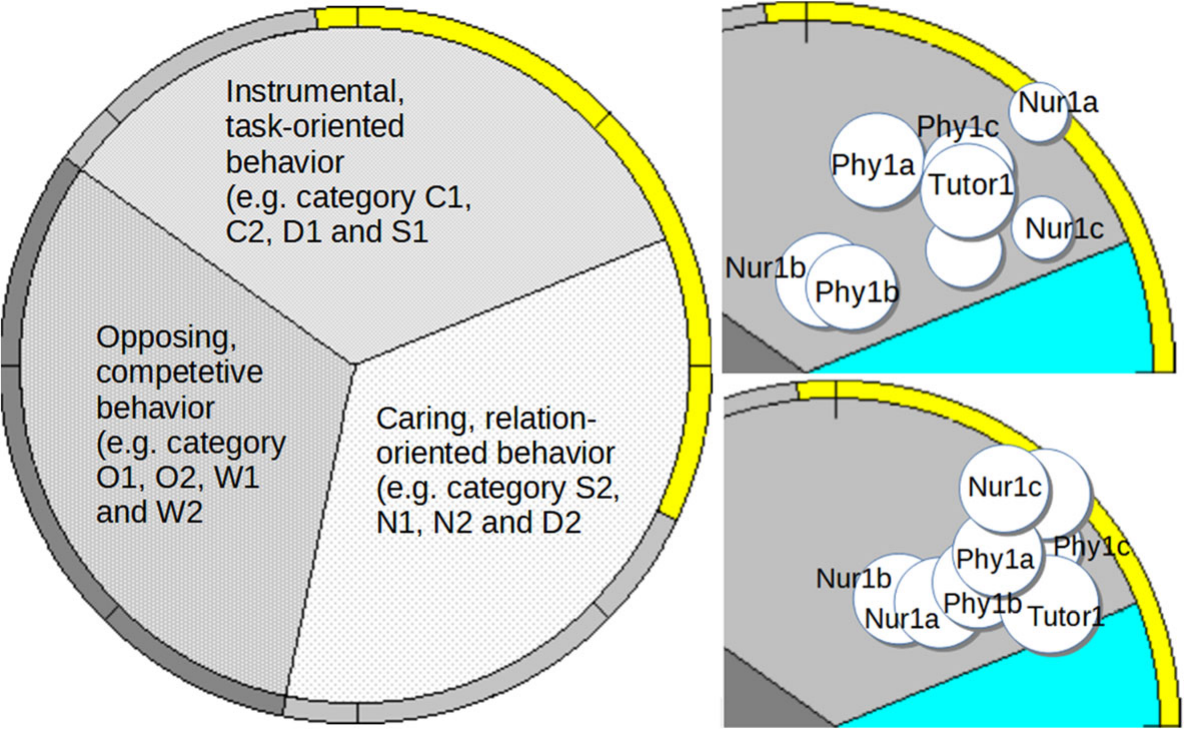
**a**, **b**, **c** Tutorial group 1 with complete field diagram

**Fig. 2:**
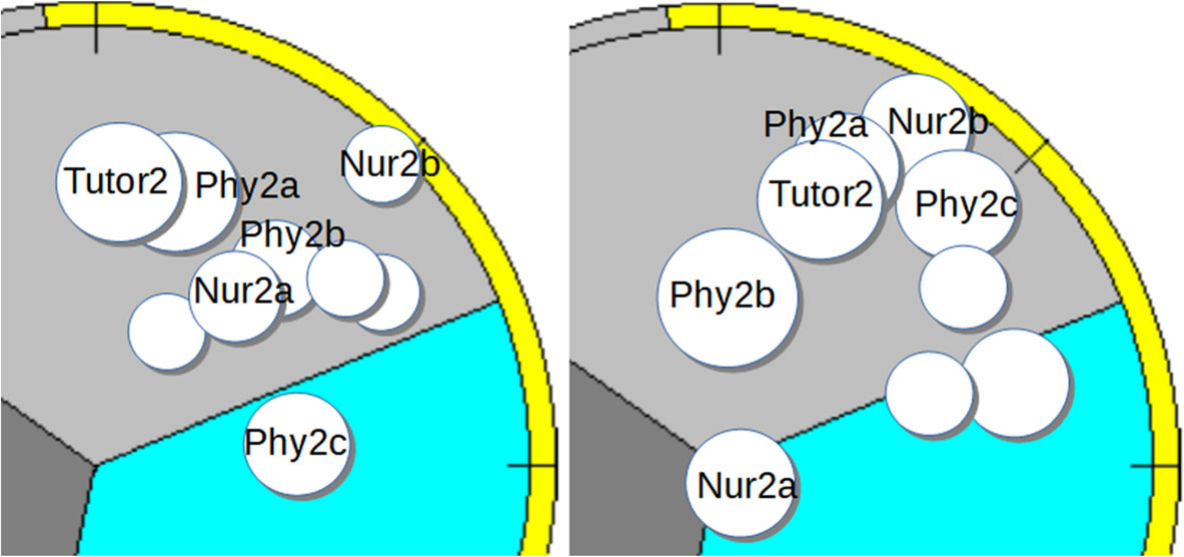
**a**, **b** Tutorial group 2

**Fig. 3:**
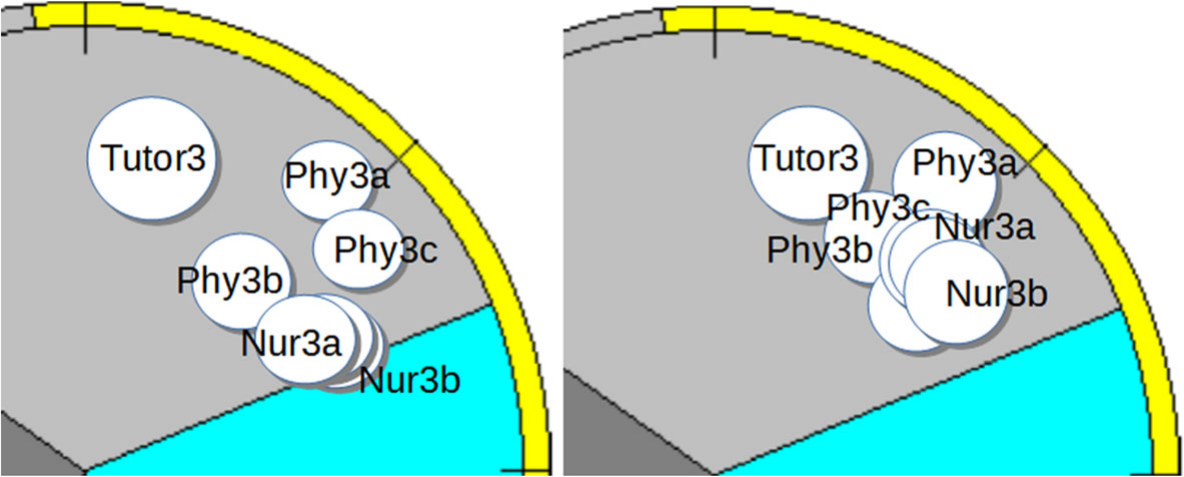
**a**, **b** Tutorial group 3

**Fig. 4:**
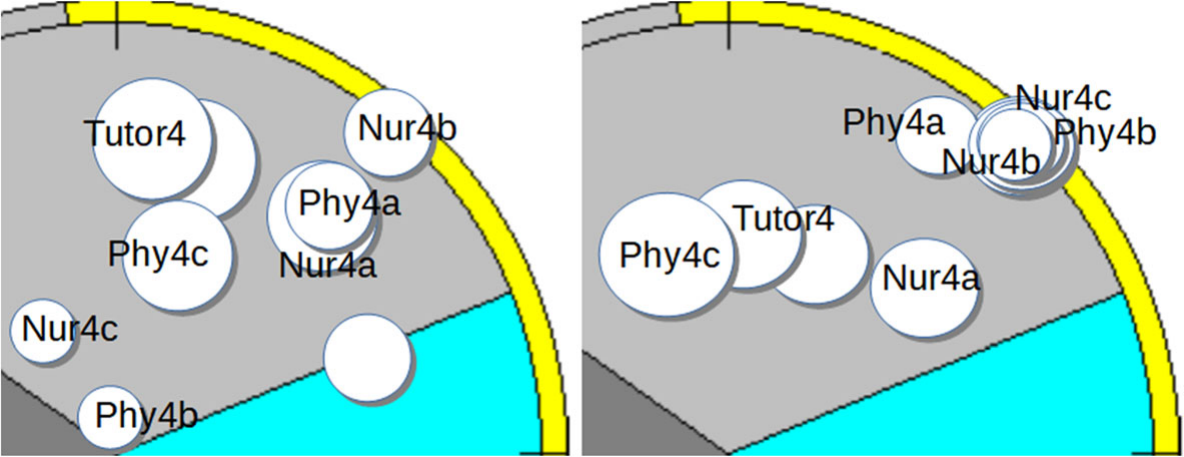
**a**, **b** Tutorial group 4

The data are analyzed post-observation using SPGR algorithms, which produce both statistics and the group’s dynamic (Fig. 1a,b,c to 4a, b). These analyses and diagrams let us evaluate the role structure, represented by the position of each member in the social field, and the distribution of influence in the group using the circle size of each person. Analysis of inter-rater reliability on a 10% sample of the data showed an acceptable level of agreement (69%). The exact requirement for acceptable consistency in direct observations depends on the nature of the study. In the present study, the level of inter-rater reliability is based on each observation, including tree decisions, and thereby an acceptable level of agreement and considered good. A low level of agreement would indicate a lower level of reliability and credibility, probably resulting in an incomplete study. The study also has high face validity as both researchers involved in the observations subjectively and independently recognized the different tutorials when seeing the field diagrams.

## Results

There were four groups in this study, with a group size varying from six to nine students. All groups had three physicians and the number of nurses varied from two to three (Table 1). The rest of the members came from other healthcare professions, such as physiotherapy, occupational therapy and speech therapy. Each group had a tutor. Over the six-week period, the activity of all four tutorial groups was dominated by task-oriented behavior, but their group-dynamics differed and their development over the observed period took different paths. Groups 1 and 3 (Figs. 1a,b,c and 3a, b) became more cohesive, whilst Group 2 (Fig. 2a, b) became more fragmented and Group 4 (Fig. 4a, b) became more polarized.

In Figs. 1b,c to 4a,b each person is mapped in SPGR field diagrams. Each person is marked by a circle. If the person shows mainly task-oriented behavior, the circle is positioned in the upper sector of the diagram, mainly caring behavior in the lower right sector and mainly opposing behavior in the lower left sector. Larger circle sizes indicate dominant behavior. The left field diagram represents observations from the first group session and the right diagram from the last.

### Tutorial group 1

Tutorial group 1 consisted of one tutor and seven students; three physicians (Phy1a, Phy1b and Phy1c), three nurses (Nur1a, Nur1b and Nur1c), and one other healthcare profession (Fig. 1b,c).

To the left in Fig. 1 a complete field-diagram is presented. Instrumental and task-oriented behaviors appear in the upper sector, relation-oriented behavior in the lower right sector and opposing behavior in the lower left sector. Based on how much of certain behaviors a person shows, he or she will be placed accordingly in the diagram. Members of fairly high functioning groups appear in the upper right section of the diagram, which is the case for the groups in this study. Each circle represents one group-member. The top right diagram (1b) is from the start of the six-week period and the bottom right (1c) from the end. The circles labelled Phy represent physicians and Nur represent nurses. This applies throughout. In the right bottom diagram the circles are closer together, indicating that this group became more cohesive over the six-week period. Distribution of influence, illustrated by circle size, also became more equal among team-members as seen in the diagram to the right.

#### The first tutorial group session

The predominant behavior of all members was task-oriented (located in the upper sector of the diagram). The group was somewhat fragmented. Nur1b and Phy1b join in a subgroup toward the opposing sector (lower left). Phy1a and Tutor1 are the most dominant members (largest circle size), while Nur1a and Nur1c are the most submissive. The position of Phy1c and Tutor1, with balanced task-oriented and dominant behaviors, indicates that they played leading roles and were willing to support other group members. For example, when Nur1c had trouble starting an online contact group, Phy1c immediately presented his/her laptop and offered to help; Phy1c: “If you access this page... wait, I’ll show you.” This is in contrast with Nur1b’s reaction when Nur1c was late for the session; Nur1b: “Showing up on time is important, isn’t it?” and Nur1c’s reaction: a gentle smile, downward gaze and closed body-language. Tutor1 mostly used non-verbal behaviors such as nodding and consistently looking at whoever was speaking but, as her large circle indicates, this had a significant influence on the group.

#### The last tutorial group session

During this session, the group displayed more cohesion, with all members contributing more equally (larger and equally sized circles) to the task. Phy1b has moved from opposition to show supportive behavior, albeit in a submissive manner. Nurs1c had moved from being submissive to the most enthused. Tutor1 is not as much in charge and instead plays a highly supportive role.

#### Summary of the tutorial Group’s development

Over the 6 weeks of interprofessional work, the tutorial group had evolved into a more cohesive group and the initial pattern of subgroups and fragmentation had dissipated. All the nurses acted according to their professional stereotypes; being fairly submissive in the first session and more balanced and active in the last. The entire group moved slightly towards the lower right sector, representing more relationship-oriented behavior.

### Tutorial group 2

Tutorial group 2 consisted of one tutor (Tutor2) and eight students. Three physicians (Phy2a, Phy2b and Phy2c), two nurses (Nur2a and Nur2b) and three students from other healthcare professions. The averages of observed behavior for each person in the two sessions are presented in Fig. 2a, b.

This group was fragmented at the start with a dominating tutor and became even more fragmented at the end of the six-week period (right diagram).

#### The first tutorial group session

Two members, Tutor2 and Phy2a, show strong task-orientation and dominance, polarized to the other members that are rather submissive (smaller circle sizes). Phy2c is the only one showing mostly relation-oriented behavior and is the one farthest away from the group. All physicians showed behavior more dominant than the nurses and strongly influenced the group’s work, again consistent with their professional stereotypes. This is illustrated by an interaction during the meeting between Phy2a and Phy2c. After some back and forth discussion of when individual contributions should be delivered, Phy2a stated firmly “You who’ve got children, just upload your work when the children are in bed”. Phy2c replied with a gentle smile: “We are just talking about one or two pages, not a thesis.” When evaluating the group’s work, Phy2c stated: “Some have worked more than others!” which was confirmed by another member: “Yes, it feels like we’ve let the others do the job for us.” Tutor2 was rather authoritarian, for example when telling the group what to include in their texts: “There must be some facts in some way. But you do understand that it can’t be 17 pages, it gets far too long.” Interactions in the group are best described as fragmented, with several members appearing withdrawn and evasive.

#### The last tutorial group session

In this session, the group dynamic was quite different. Phy2a and Tutor2 still have dominating roles but are now accompanied by Nur2b and Phy2c. Phy2c’s nurturing role in the first session is now handled by two others in combination. Phy2b and especially Nur2a are now in opposition to the rest of the group, and the group is even more fragmented than in the first session.

#### Summary of the tutorial Group’s development

The fragmentation and tendency to polarization that were observed in the first session emerged more clearly in the last session. Over the intervening 6 weeks, all members have become more active, but not more collaborative and two members (Phy2b and Nur2a) are now in opposition.

### Tutorial group 3

Tutorial group 3 (Fig. 3a, b) included two tutors (one in the first session and one in the last session) and six students: three physicians (Phy3a, Phy3b and Phy3c), two nurses (Nur3a and Nur3b) and one other healthcare professional. The tutors are labeled Tutor3 in both cases.

As with group 1, this group became more cohesive over the period. The tutor show dominating and authoritarian behavior like the tutor of group 2. Also, for this group, the distribution of influence (circle size), became more equal among team-members over time (right diagram).

#### The first tutorial group session

All members showed task-oriented but submissive behavior, except Tutor3 who was both dominant and demanding. For example, when Phy3a asked: “Should we decide on our topic now?” Tutor3 replied firmly: “No, we are still brainstorming!” instead of throwing the question back to the group to encourage them to reflect on their own process and collaboration. Group 3 may be labeled a “strong leader – follower” group. This is seen in Fig. 3a, b, which shows one large circle around Tutor3a, with the other members represented by small circles (submissive behavior).

#### The last tutorial group session

In this session, Phy3a and Phy3b played more active roles and all members contributed more equally. For example, when the group talks about how the social service demands that healthcare should promote social participation and Phy3b says: “I think that 30 minutes with someone cooking for him [the patient] would make a difference in many ways.” The members agree, followed by a series of suggestions on how healthcare could promote social participation. Although Tutor3 still showed firm and dominating leadership, the group had become more cohesive.

#### Summary of the tutorial Group’s development

In the first session, the group consisted of a strong leader, Tutor3, and followers. By the last session, the group had become more cohesive with more equally contributing members, even though Tutor3 remained authoritarian.

### Tutorial group 4

Tutorial group 4 (Fig. 4a, b) consisted of a tutor (Tutor4) and nine students: three physicians (Phy4a, Phy4b and Phy4c), three nurses (Nur4a, Nur4b and Nur4c) and two other healthcare professionals.

Group 4 started out as a fragmented group with an authoritarian and dominating tutor (left) and ended as a polarized group (right) with two subgroups; one with members Phy4c, Tutor and one other member, and one with members Phy4a, Phy4b, Nur4b, Nur4c and one other. Nur4a is partly outside the first sub-group and may act as a mediating role in the polarization.

#### The first tutorial group session

Most members showed mostly task-oriented behavior. Tutor4, Phy4c and another member formed a leading coalition, while Phy4b and Nur4c show passive opposition. A subgroup of followers (Nur4a, Phy4a and Nur4b) was somewhat in opposition to the leading coalition, with Nur4a in a leading role (the largest circle). An example of submissive behavior occurred when Phy4a said to another student, who was standing writing at the board, in a very quiet voice, that the message needed to be repeated. An example of passive opposition is Nur4c’s constant checking of his/her mobile phone whilst the others were involved in discussion.

#### The last tutorial group session

In this session, a change of the group’s dynamics was evident, with even stronger polarization between two more distinct sub-groups. One dominant and opposing sub-group (Nur4a, Phy4c, Tutor4 and another) versus a cohesive and submissive sub-group (Phy4a, Nur4b, Nur4c, Phy4b and one other), trying its best to be supportive and encouraging. A typical example of communication between the sub-groups occurred during the discussion of whether a patient with dementia should drive a car or be offered a travel service. Phy4c: “We do not know enough; we only have his [the patient’s] statement.” Nur4a: “What should we do then?” Phy4c “There is a test with different scales that are recommended...”.

#### Summary of the tutorial Group’s development

During the 6 weeks, tutorial group 4 developed from a fragmented and somewhat polarized group to a strongly polarized group, with an opposing and dominant subgroup towards a cohesive and submissive sub-group committed to task-oriented collaboration. Tutor4 was part of the dominant sub-group in both sessions.

In Table 3 the key points of the result are summarized for each tutorial group’s development based on group-dynamics, collaboration, and tutor style.

**Table 3:** Summary of the key results regarding the effect of group-dynamics, collaboration and tutor style for each tutorial group

Tutorial group	Group development; Group-dynamics, influence, collaboration & tutor style
**Tutorial group 1**	• from fragmented to more cohesive group dynamics • develops a more equal influence and interprofessional collaboration • stereotyped profession-based behavior became less frequent • mediating tutor style;
**Tutorial group 2**	• from somewhat fragmented to more fragmented group dynamics • develops a more equal influence but due to dominating students reduced quality of interprofessional collaboration • stereotyped profession-based behavior became less frequent • authoritarian tutor style, is a part of a dominant sub-group
**Tutorial group 3**	• from fragmented to more cohesive group dynamics • develops a more equal influence and interprofessional collaboration • solid remaining non-stereotyped profession-based behavior • authoritarian tutor style
**Tutorial group 4**	• from somewhat polarized to strongly polarized group dynamics • develops a more equal influence but due to dominating sub-groups not the interprofessional collaboration in the tutorial group overall • reduced quality of interprofessional collaboration • stereotyped profession-based behavior became less frequent • authoritarian tutor style, is a part of a dominant sub-group

## Discussion

There is a growing demand for iPBL as a way to introduce interprofessional collaboration among pre-qualified healthcare professionals who are still completing their education [4–6]. The goal is to enhance collaborative practice skills among learners with the expectation that this may eventually lead to improved patient safety and patient care [12, 24]. There seems to be overall agreement that iPBL is effective and that it fosters collaborative practice [4, 5, 26]. One large university in Sweden has adopted this approach, with students from different healthcare programs learning together in interprofessional tutorial groups [9, 13, 25] using PBL [2]. This raises questions of group processes and whether they are influenced by physician and nurse profession-based stereotypes, as well as by the tutor’s role over sessions in inter-professional tutorial groups in PBL. This study examined group processes and leadership in interprofessional tutorial groups, an under-investigated field of research [2, 5, 19].

### Four tutorial groups –two patterns of group development

The group dynamics in all four tutorial groups changed over the 6 weeks of collaboration, while following different paths. Two of the groups (tutorial groups 1 and 3) became more cohesive with a higher quality of intra-group communication, whereas group 2 and 4 became, respectively, more fragmented and polarized [43, 47], suggesting a reduced quality of collaboration. The distribution of influence among group members is an important parameter of group dynamics. All groups showed a more equal distribution of influence in the second session, and stereotypical behavior became less frequent. In these two aspects all groups improved, indicating that interprofessional collaboration took place [4–6, 10, 16]. Group 1 became less fragmented with a more even distribution of influence by the last session, whilst the pattern of activity in group 2 remained unchanged. Groups 3 and 4, on the other hand, becoming even more task oriented. Tutorial group 1 showed in addition less opposing behavior, while tutorial group 2 showed more opposition. Members of tutorial group 1 also increased their contribution over the 6 week period. Combined with a more equal distribution of influence than the other groups, we can conclude that the developmental trajectory of this group was superior to those of the other three groups [43, 47].

### Stereotypical behaviors

Three of the four tutorial groups showed more professionally stereotypical behaviors in the first session than the last. In session 1, the physicians in groups 1, 2 and 4 dominated (18, 30] and behaved in a way that is consistent with physician stereotypes [29, 32–34]. The nursing and other healthcare students all behaved submissively in the first group session, consistent with the stereotypical role of nurses [15, 17, 20, 26, 50]. In the last group session, the physicians no longer dominated; the influence of the other students had increased. One interpretation is that during the first session, the physicians in groups 1, 2, and 4 presented a positive example that inspired collaboration and encouraged all group members to engage [11, 15, 29, 50]. In addition, a few physicians were more submissive during the 6-week course. Tutorial group 3 showed a different pattern, as all the students were submissive in session 1. The tutor alone behaved flexibly and dominated the group. In the last group session, however, almost all group members behaved more flexibly, hence promoting beneficial collaboration.

Our results are in accordance with previous research showing that stereotypical professional behavior and preconceived notions about one’s chosen profession and its allied professions are deeply rooted and difficult to change [20, 27, 30, 50]. Being locked into professionally stereotypical behavioral patterns may have a negative impact on interprofessional collaboration, suggesting that not all group members’ competences are fully exploited [15, 16, 29, 49]. Neither the development nor the performance of such groups is optimal. Our findings suggest, however, that iPBL may loosen professional stereotypes (e.g. physicians become less dominant and nurses less submissive).

### iPBL

One of the premises of spin theory is that a high-functioning group should be able to adapt to different contexts and tasks [43, 47]. This aligns well with iPBL, which is a student-centered, context-dependent approach to learning [3, 13, 19] a point further emphasized by Freeth et al. [5]. Hence, being a student-centered, problem based, self-directed learning context in which the interprofessional tutorial groups foster learning collaboration, the utilization of all members’ competences and contributions, problem-solving, and reflection are natural components of the work of iPBL groups [9, 11, 19]. The students who participated in this study were used to PBL, but what was new to them was the interprofessional composition of the tutorial groups. One interpretation of the results is that the iPBL method played a role in the breaking down of professional roles and increasing behavioral flexibility during the six-week course. This is in line with previous research suggesting that iPBL is more naturally suited to increasing collaboration between pre-qualified students from different healthcare specializations [4, 5] but this is something that needs further investigation.

### The effect of the Tutor’s role on the success of iPBL

Even though we argue that iPBL is a good way of breaking down professional boundaries, it is clear that the effects are strongly influenced by the tutor’s role and behavior [2, 3]. In all groups, the tutor was clearly active, dominating, directing and setting the agenda for the group’s work. Whilst this may not, at first sight, appear consistent with the principles of iPBL, it reflects the principle that tutors should facilitate the processes of the group rather than deliver content (11, 19, 35]. The chances of a successful group dynamic evolving are maximized if the tutor’s role and behavior is negotiated and developed in interactions with members of the group and adapted to the context.

In one group (tutorial group 1), the tutor showed significantly more mediating behavior than the tutors in the other three. This group followed the most positive developmental trajectory, becoming more cohesive and more self-managing, with members having more similar influence in the last session than the first. By the last session, the tutor had moved from a task-oriented role to a more supportive and caring one, which is consistent with a desirable development of the tutor’s role in iPBL [3, 19, 36, 37]. In all the other groups, the tutor initially had a more directing and authoritarian facilitation style that endured and was still clearly evident in the last session.

None of the other groups were as successful as group 1, although group 3 had a fairly positive developmental trajectory. The members of group 3 were all rather submissive and although the group became more cohesive, the tutor’s task-oriented behavior persisted. The difference between group 3 and groups 2 and 4 was that, in the latter groups, the authoritarian tutor was part of a dominant sub-group in session 1. We argue that this association is the main reason for the negative development of these two groups. Whilst we argue that iPBL is a good way of breaking down stereotypical behavior in groups, we also argue that the tutor’s role is a vital factor in the success of the approach. Knowing the effect of how group-processes can enhance mutual understanding across pre-qualified students in healthcare education has important implications for further development of courses using iPBL. Paying attention to students’ development of academic knowledge, self-directed learning, problem solving abilities and collaborative skills gives tutors practical tools for supporting and reflecting on the group’s work and process. In addition, tutors become better able to challenge members to develop and apply metacognitive skills and interprofessional collaboration. One significant implication for tutors is that their enhanced knowledge of how iPBL can reduce stereotypical behaviors and positively increase members’ ability to engage in inter-professional collaboration, might influence how they perform their roles as tutors. Since tutor style significantly influences the development of iPBL groups’, a potential challenge is to find competent tutors who are interested in developing mutual understanding both across pre-qualified students and different health care professions.

### Limitations

The main limitations of this study are the small number of groups and that the analysis is based on observations of just 4 hours of video recording. Ideally, we would enlarge our study and include more groups “but that does not justify ignoring the information we could obtain” [51]. Using natural groups in an authentic situation affects the availability of participants. As the focus in this article is group dynamics and collaboration, another limitation concerns the analysis focusing on physicians, nurses and “other healthcare professionals.” Due to a small number of participants from other healthcare professions (physiotherapy, occupational therapy and speech therapy) they are merged into one group. While not neglecting the fact that all participants in the tutorial group influence the group-dynamics, and accordingly the pattern of collaboration, this grouping was done to secure participants’ confidentiality. However, an in-depth analysis in which each profession is analyzed individually shows an agreement with the results presented in this article.

The level of inter-rater reliability (69%) might be considered a limitation but given that each observation was actually based on three decisions (who acts towards whom, category, and verbal or non-verbal; see Method section), as described above in the group behavior section on the SPGR system for direct observations. We argue that despite these limitations, the analysis of a small sample can lead to interesting insights.

Another limitation concerns the sparse information on an individual level. This limitation is due to ethical restrictions for this particular study. However, as the focus in this study is on group processes in iPBL and the tutorial groups are authentic groups in their natural setting the study contributes with significant information about group phenomena which is transferable to other iPBL environments [52, 53].

### Future research

Given the limited number of groups in the present study, replications with PBL groups in the same or similar contexts with more extensive observation would be valuable. Although we can draw some practical implications for conducting interdisciplinary PBL groups from this study, a broader base of research will increase the validity of such advices. Group-observation as used in this study, demands a trained observer and a heavy workload. We will point to research using sensor technology for automated interaction-analysis for developing practical tools to analyze PBL group-dynamics.

## Conclusion

The purpose of this study was to investigate whether changes in group processes, collaboration, and tutor style, influence the perception of profession-based stereotypes of physician- and nursing-students. We found a clear pattern of behavior reflecting professional stereotypes of nurses and physicians in the initial group observation, and all four tutorial groups changed their group’s dynamics over the 6 weeks of the course. Important elements of group-dynamics such as distribution of influence, polarization, and opposition behavior changed, but the pattern of group development took different forms. Two of the groups became more cohesive, one more fragmented, and one more polarized.

Our findings suggest that stereotypical professional behaviors are already present among students of different health-related professions. Physicians’ classically dominant position versus the more submissive approach associated with nurses were observed in the initial session of this study. However, in the last sessions, when groups had been working together for 6 weeks, stereotypic behaviors became less frequent. We argue that iPBL forces students to see the person behind the profession rather than the profession-based stereotype. An important aspect of iPBL is that all members must contribute equally for the problem to be solved. Whether it is the form of working and learning in small interprofessional composed groups or the problem-based pedagogy that helps reduce the negative profession-based stereotypes is beyond the scope of this article, but further research might answer that question.

Apart from observing fewer professional stereotypes in the second observation, we found that tutor’s behavior strongly influences group-development. The groups in which the tutor’s role was consistent with the principles of PBL showed an improvement in group-dynamics and collaboration.

## Data Availability

The data generated and analysed during the current study are not publicly available due to existing ethical agreements in the project, participants’ anonymity and confidentiality, and out of respect for the participants’ sensitive contribution. Data are however available from the authors upon reasonable request.

## Abbreviations

IPBL: Inter-professional Problem-Based Learning
IPE: Interprofessional Education
Nur: Nurse
PBL: Problem-based learning
Phy: Physician
SPGR: Systematizing the Person Group Relation
C: Control
C1: Ruling
C2: Task-oriented
N: Nurture
N1: Caring
N2: Spontaneous
O: Opposition
O1: Criticism
O2: Assertiveness
D: Dependence
D1: Loyalty
D2: Acceptance
S: Synergy
S1: Engagement
S2: Empathy
W: Withdrawal
W1: Resignation
W2: Selfish

## References

[1] Barrows H (1986). A taxonomy of problem-based learning. Med Educ.

[2] Öystilä S, Poikela E, Raija Nummenmaa A (2006). The significance of group dynamics in problem-based learning. Understanding problem-based learning.

[3] Wiggins S, Hammar Chiriac E, Larsson Abbad G, Pauli R, Worell M (2016). Ask not only ‘what can PBL do for psychology?’ But ‘what can psychology do for PBL?’ A review of the relevance of problem-based learning for psychology teaching and research. Psychol Learn Teach.

[4] Abu-Risch E, Kim S, Lapio C, Varpio L, Malik E, White AA, Craddick K, Blondon K, Robins L, Nagasawa P, Thigpen A, Chen L-L, Rich J, Zierler B (2012). Current trends in interprofessional education of health sciences students: a literature review. J Interprof Care.

[5] Freeth D, Savin-Baden M, Thistlethwaite J, Swanwick T, Forrest K, O’Brien B (2019). Interprofessional education. Understanding medical education: evidence, theory and practice.

[6] Vyt A, Pahor M, Tervaskanto-Maentausta T (2015). Interprofessional education in Europe: policy and practice.

[7] Barr H (2002). Interprofessional education: today, yesterday and tomorrow.

[8] CAIPE (2019). Welcome to CAIPE: The Centre for the Advancement of Interprofessional Education.

[9] Lindh Falk A, Dahlberg J, Ekstedt M, Whiss P, Heslyk A, Abrandt Dahlgren M, Vyt A, Pahor M, Tervaskanto-Maentausta T (2015). Creating spaces for interprofessional learning: strategic revision of a common IPL curriculum in undergraduate programmes. Interprofessional education in Europe: policy and practice.

[10] Thistlethwaite J (2012). Interprofessional education: a review of context, learning and the research agenda. Med Educ.

[11] Jewell L, D’Eon M, McKee N, Proctor P, Trinder K (2013). Tutor experiences with facilitating interprofessional problem-based learning. J Res Interprof Pract Educ.

[12] World Health Organization (2010). Framework for action on interprofessional education and collaborative practice.

[13] Wilhelmsson M, Pelling S, Ludvigsson J, Hammar M, Dahlgren L-O, Faresjö F (2009). Twenty years of experience of interprofessional education in Linköping: groundbreaking and sustainable. J Interprof Care.

[14] Gudmundsen A, Norebye B, Abrandt Dahlgren M, Obstfelder A (2019). Interprofessional student meeting in municipal health service – mutual learning towards a Community of Practice in patient care. J Interprof Care.

[15] Adams K, Hean S, Sturgis P, Macleod Clark J (2006). Investigation of the factors influencing professional identity of first-year health and social care students. Learn Health Soc Care.

[16] Sollami A, Caricati L, Mancini T (2018). Attitudes towards interprofessional education among medical and nursing students: the role of professional identification and intergroup contact. Curr Psychol.

[17] Foronda C, MacWilliams B, McArthur E (2016). Interprofessional communication in healthcare: an integrative review. Nurse Educ Pract.

[18] MacArthur BL, Dailey SL, Villagran MM (2016). Understanding healthcare providers' professional identification: the role of interprofessional communication in the vocational socialization of physicians. J Interprof Educ Pract.

[19] Wiggins S, Abrandt Dahlgren M, Ekstedt M, Hammar Chiriac E, Törnqvist T, Bridges S, Imafuku R (2020). The first PBL tutorial: breaking the ice: how students present themselves to the group in an interprofessional problem-based learning context. Interactional research into problem-based learning.

[20] Bell AV, Michalec B, Arenson C (2014). The (stalled) progress of interprofessional collaboration: the role of gender. J Interprof Care.

[21] Lorentz W (2009). Europe, the professions and interprofessional education: an exploration in inter-culture relativity. J Interprof Care.

[22] Imafuku R, Kataoka R, Mayahara M, Suzuki H, Saiki T (2014). Students’ experiences in interdisciplinary problem-based learning: a discourse analysis of group interaction. Interdisc J Problem Based Learn.

[23] Dahlgren LO (2009). Interprofessional learning and problem-based learning: a marriage made in heaven?. J Interprof Care.

[24] Faresjö T, Mogensen E, Ponzer S (2009). Framtidens vård kräver interprofessionellt samarbete [future care requires interprofessional collaboration]. Läkartidningen.

[25] Wilhelmsson M (2011). Developing interprofessional competence: theoretical and empirical contributions.

[26] Ateah AA, Snow W, Wener P, MacDonald L, Metge C, Davis p., Fricker, M., Ludwig, S., & Anderson, J. (2011). Stereotyping as a barrier to collaboration: does interprofessional education make a difference?. Nurse Educ Today.

[27] Thylefors I (2013). Babels torn: om tvärprofessionellt teamsamarbete. [Babylon's tower: about cross-professional teamwork].

[28] Blomqvist S (2009). The utilization of competence in multi-professional psychiatric teams.

[29] Sjøvold, E., & Hegstad, A.-C. (2008). Group dynamics, professional stereotypes and dominance: the performance of interdisciplinary teams in hospitals. In S. Jern & J. Näslund (Eds.), Dynamics within and outside the lab: proceedings from the 6th GRASP conference, Lund University, may 2008 (159–183). Sweden: Lund University, POST Network, Lund University.

[30] Frost HD, Regehr G (2013). “I AM a doctor”: negotiating the discourses of standardization and diversity in professional identity construction. Acad Med.

[31] Traynor M, Buus N (2016). Professional identity in nursing: UK students’ explanations for poor standards of care. Soc Sci Med.

[32] Bell L, Allain L (2011). Exploring professional stereotypes and learning for inter-professional practice. An example from UK qualifying social work education. Soc Work Educ.

[33] Carpenter J (1995). Doctors and nurses: stereotypes and stereotype change in interprofessional education. J Interprof Care.

[34] Pietroni P (1991). Stereotypes or archetypes? A study of perceptions amongst health-care students. J Soc Work Pract.

[35] Azer S (2005). Challenges facing PBL tutors: 12 tips for successful group facilitation. Med Teach.

[36] Hammar Chiriac E, Näslund J, Östergren M-L (2010). Basgruppshandledning inom PBL på högskola och universitet [tutorial group supervision within PBL at higher education and university]. Grupphandledning. Forskning och erfarenheter från olika verksamhetsområden.

[37] Dolmans D, Wolfhagen I, van der Vleuten C, Wijnen W (2001). Solving problems with group work in problem-based learning: hold on to the philosophy. Med Educ.

[38] Rosander M, Hammar Chiriac E (2016). The purpose of tutorial groups: social influence and the group as means and objective. Psychol Learn Teach.

[39] Ancona D, Bresman H (2007). X-teams.

[40] Edmondson AC (2012). Teaming.

[41] Healey MP, Vuori T, Hodgkinson GP (2015). When teams agree while disagreeing: Reflexion and reflection in shared cognition. Acad Manag Rev.

[42] Pentland A (2014). Social physics.

[43] Sjøvold E (2014). Resultater gjennom team. [results through teams].

[44] Sjøvold E (2007). Systematizing person-group relations (SPGR): a field theory of social interaction. Small Group Res.

[45] Björnstjerna Hjelm A. Inte så stereotypt som man kan tro - om interprofessionellt sammansatta basgruppers utveckling vid problembaserat lärande. [not as stereotype as one can believe -about interprofessional composite base groups development in problem-based learning]. [Master’s thesis]. Linköping: Linköping University; 2017.

[46] Hammar Chiriac E, Einarsson C. Gruppobservationer. Teori och praktik (tredje upplagan) [Group observations. Theory and practice. 3rd ed.] Lund: Studentlitteratur; 2018.

[47] Sjøvold E. Teamet: Utvikling, effektivitet og endring i gruppe. [Team: Development, efficiency and change in groups.]. Oslo: Universitetsforlaget; 2006.

[48] Sjøvold E (2002). SPGR manual.

[49] Sjøvold E, Andre B (2020). Interprofessional stereotypes in physician and nurse education in Norway (work in progress).

[50] Braithwaite J, Clay-Williams R, Vecellio E, Marks D, Hooper T, Westbrook M, Ludlow K (2016). The basis of clinical tribalism, hierarchy and stereotyping: a laboratory-controlled teamwork experiment. BMJ Open.

[51] Mercer N (2008). The seeds of time: why classroom dialogue needs a temporal analysis. J Learn Sci.

[52] Bryman A (2015). Social research methods [5th edition].

[53] Lincoln YS, Guba E (1985). Naturalistic inquiry.

[54] British Psychological Society (2014). Code of human research ethics.

